# Factors influencing the tobacco control policy process in Egypt and Iran: a scoping review

**DOI:** 10.1186/s41256-017-0039-6

**Published:** 2017-07-10

**Authors:** C. Banks, S. Rawaf, S. Hassounah

**Affiliations:** WHO Collaborating Centre for Public Health Education & Training, Department of Primary Care & Public Health, School of Public Health, Imperial College London, Charing Cross Campus, 3rd Floor, The Reynolds Building, St Dunstan’s Road, London, W6 8RF UK

**Keywords:** Public health, Health policy, Prevention strategies, Tobacco control, Implementation science

## Abstract

**Introduction:**

Tobacco control policy is essential for addressing the growing tobacco consumption seen in the Eastern Mediterranean Region, the single greatest preventable contributor to the non-communicable disease epidemic. Egypt and Iran have had varied success in using policy to combat this issue. The study aims to identify and compare the factors which have influenced different stages of the policy process – evidence generation, development and implementation.

**Methods:**

A scoping review was conducted with a systematic search of 7 databases which was conducted along with searches of Google Scholar, and the World Health Organisation and Eastern Mediterranean Regional Office websites to identify influencing factors at each stage of the policy process.

**Results:**

Twenty-seven relevant articles were identified from the literature search. Factors identified as influencing tobacco control policy in these countries were lobbying by the tobacco industry, the rise of water-pipe smoking, lack of political commitment and the lack of resources to for policy implementation. Iran was found to be leading Egypt on all three areas of the policy process. Implementation was found to be the most pivotal part of the policy process and the area in which Egypt was weakest compared to Iran.

**Conclusion:**

This study addresses a gap in knowledge concerning tobacco control in the Middle East and has identified multiple factors which are potentially slowing the process of enforcing policy to address tobacco consumption. Iran is the regional leader for tobacco control and it is important for Egypt to assess the transferability of its tactics and immediately start implementing measures to control tobacco use.

## Background

Tobacco consumption is the leading single cause of preventable death globally. It is estimated to have caused up to 100 million deaths in the 20^th^ century, and without significant intervention could kill up to 1 billion in the 21^st^ century [1]. The global prevalence of tobacco users in 2012 was estimated at 21% of all over 15 year olds (1.1 billion people) by the World Health Organisation (WHO), between a half and two thirds of whom will die from smoking related conditions [2–4]. Although global prevalence has fallen in the last few decades, the absolute number of smokers has increased by 246 million since 1980 due to population growth [5]. This presents a major threat to global development as the health burden associated with smoking causes substantial loss of economic productivity, particularly as over 80% of current tobacco users live in low and middle income countries (LMIC) [3]. Projected figures for 2030 state that deaths resulting from tobacco use will increase from 6 million in 2014 to 8 million annually, 80% of these in LMIC.

During the 20^th^ century, a shift was observed in the demographics of smoking. Consumption of tobacco decreased in the second half of the century in most high income countries (HIC), which was then followed by a decline smoking related in death in these countries [5, 6]. Meanwhile, tobacco consumption has been increasing in LMIC as the tobacco industry moves its focus for marketing and promotional efforts in order to exploit weaker regulation and lower awareness of the risks [1, 7]. This combination has resulted in the tobacco industry being able to employ tactics as it chooses, particularly as 40% of LMIC did not have any tobacco advertisement bans in 2012 [8].

The consumption of tobacco products is a rapidly growing problem in the Eastern Mediterranean Region (EMR) [1]. Egypt and Iran are two of the most influential countries in the EMR as well as being among the largest and similar in size, with comparable population demographics [9]. Gross domestic product (GDP) per capita is higher in Iran at $5,442.9 compared to $3,198.7 in Egypt [10]. While smoking prevalence is markedly higher in Egypt for males, with 47.5% of over 15 year olds smoking as opposed to 22.4% in Iran, there are cultural similarities in smoking trends [11]. Both countries have developed extensive tobacco control policy in recent years and were first (Iran) and joint second (Egypt) in a recent comparison of policies in the EMR [12]. However, there is a very apparent inconsistency between the prevalence of smoking in Egypt and Iran, with Egypt having higher smoking rates than Iran in both sexes and at all age groups [11].

Both countries had signed and ratified the WHO’s Framework Convention on Tobacco Control (FCTC) by the end of 2005, showing their commitment to using policy to control the tobacco epidemic [13]. Progress has, however, been dissimilar in the two countries, and investigating the contextual factors which may have influenced this allow the development of a roadmap to replicate success and try different solutions as used elsewhere.

Ensuring that policy has impact is a complex process and dependent on its journey from evidence generation and research, through the stage of policy formulation and development, to implementation, and it is vital that bridges are built between these essential components. For the purpose of this review, the three stages of evidence generation, development and formulation, and implementation have been drawn out as distinct steps as inferred by the WHO’s Introductory to Tobacco Control legislation [8]. Regarding tobacco control policy, these three stages have been repeatedly singled out.

Research and surveillance are central to building an evidence base for policy development. As part of the FCTC, Egypt and Iran must have comprehensive surveillance systems to monitor trends of smoking. It is then important for the research to be utilised by policy makers and used to develop feasible and effective legislation for tobacco control. Thirdly, efforts must be made to fully implement and enforce policy for it to have an impact [14]. As well as research informing the development and implementation of policy, it is imperative that evaluations regarding the implementation policy and effect are carried out in order to improve the future development of legislation [15].

The aim of this study is to identify factors influencing the process of using policy for tobacco control in Egypt and Iran, compare the role of factors in these countries and additionally, discern the level of impact these have.

## Methods

The aim of the study was addressed using a scoping review due to its multi-faceted nature and the need to identify all relevant literature regardless of study design. The Arksey and O’Malley scoping review framework, shown in Table 1, was followed [16, 17]. This method placed emphasis on a sensitive search for articles and mapping the literature found to give a comprehensive overview of the current level of research output and content, and using the literature to answer the aim of the study as best possible. This method is highly applicable to this subject area where there is a paucity of published research.

**Table 1:** Arksey and O'Malley scoping review framework

1	Identifying the research question
2	Identifying relevant studies
3	Study selection
4	Charting the data
5	Collating, summarising and reporting the results

### Identifying the research question

It was recommended by Levac et al., that the research questions for a scoping review should combine a broad question to provide breadth of coverage with a clearly articulated scope of inquiry to give direction and focus [17]. Therefore, this study aimed firstly to identify factors in the literature and then focus into whether these differed between the countries and the impact these have on the policy process.

### Identifying relevant studies

A systematic search was conducted using MEDLINE, Embase, PubMed, Global Health and Web of Science databases, and Iranian databases Scientific Information Database, Iranmedex, Magiran and Medlib. The latter country specific databases were identified through preliminary literature review which yielded relevant database sources for Iran only. Searches of grey literature were carried out on the WHO and Eastern Mediterranean Regional Office (EMRO) websites, and Google Scholar. Based on the references of the included papers, further records were included. Free text search terms included “tobacco”, “research”, “development”, “implementation”, “legislation”, “regulation”, “smok* or water?pipe”, “Egypt*”, and “Iran*”. The literature search was carried out using English, Arabic and Persian terms to maximise relevant records found. A full list of searches carried out can be found in Appendix I. The final search was conducted on December the 22^nd^ 2015. 

Three aspects of the policy process were studied: research (the process of generating evidence for policy making), development (the process of governments planning and devising policy using evidence generated through means such as epidemiological surveillance and policy evaluation), and implementation (the process of translating the policy into an operational programme in order to have an impact on the target population) [18, 19].

The literature search aimed to identify papers which contained information regarding factors which are influencing the research into, development of and implementation of tobacco control policy in Egypt or Iran.

### Charting the data

Relevant information was extracted from references by author CB. Details of articles including author, title, year of publication and design were extracted and charted using Microsoft Excel. When factors were described as having affected a stage of the policy process, this information was extracted.

### Collating, summarising and reporting the results

A scoping review intends to present an overview of all material reviewed. This review categorised the factors according to the stage of the policy process they affected and reported these as a narrative account, relaying the information extracted from the literature.

## Results

### Literature search

A total of 27 records were considered relevant to this review. Nineteen titles were included from the database literature search, an additional four titles were taken from bibliographies, and four records were added based on grey literature research. Eleven of these articles contained information relevant to Iran, 8 to Egypt, and 8 which contained information on both countries. Multiple article types were present in the included titles such as cross sectional surveys, reports using WHO data, commentaries, and reviews.

**Fig. 1:**
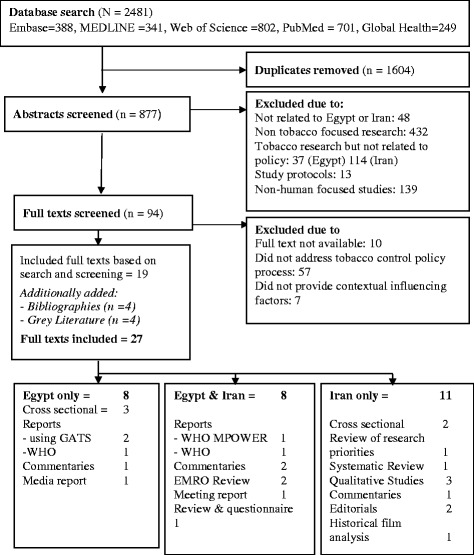
Literature review flow diagram adapted from PRISMA

The process of screening and further details on study characteristics can be found in Fig. 1, details of included references can be found in Appendix II.

### Evidence generation

#### Surveillance systems

The establishment of adequate local surveillance systems for non-communicable disease (NCD) and risk factors is critical for complementing international research and surveillance, and bridging the knowledge gap [20]. Egypt leads Iran in the number of Global Youth Tobacco Surveys (GYTS) carried out and the time span covered. Iran alone has carried out the Global School Personnel Survey (GSPS), and only Egypt has implemented the Global Adult Tobacco Survey (GATS). The Global Health Professions Student Survey (GHPSS) has been carried out in both countries on different sets of students. The WHO STEPwise approach to surveillance (STEPS) methodology for collecting standardised information on risk factors has been implemented twice in Egypt and 6 times in Iran, though neither has conducted the survey since 2012, as noted in Table 2.

**Table 2:** Dates and names of risk factor national surveillance surveys were carried out which collected information on tobacco use

	STEPS	GYTS	GSPS	GHPSS	GATS
Egypt	2005, 2012	2001, 2005, 2009, 2014		2005 Medical students	2009
Iran	2005, 2006, 2007, 2008, 2009, 2011^a^	2003, 2007	2007	2007 Dental, nursing and pharmacy	

The GHPSS results also stated which group of students they were conducted with out of medical, dental, nursing and pharmacy students. ^a^Details regarding a STEPS survey carried out in Iran in 2011 were found but the report has not been released by the WHO or the Iranian Ministry of Health

#### Institutions for tobacco research

The founding of research institutions specific to tobacco research is a factor which was repeatedly noted as having a very positive effect on research [21]. The Egyptian Smoking Prevention Research Institute (ESPRI) was launched with funding from the National Institutes of Health in the US and ran between 2002 and 2007 as funded, and aimed to carry out “*interlocking observation, intervention and policy research*” with the goal to “*add momentum to the current efforts underway to curb smoking in Egypt*” [22]. In Iran, similar institutions identified were the Respiratory Disease Network, established in 2011 so that “*public health authorities and health policy makers will collaborate with the scientific community to foster evidence based public health practices in the area of research networks*”, and the Iranian Epidemiological Association (IrEA) which focuses on the generation of evidence for policy [23, 24]. A further collaboration detailed is between the Isfahan Cardiovascular Research Centre, the Iranian Ministries of Health and Medical Education, and FCTC policymakers in 2007 [25, 26]. Iran has also joined the Global Alliance against chronic Respiratory Disease in 2008 which relays international research and knowledge [27]. As part of the Fourth Five-Year Development Plan, the Iranian Ministry of Health established an epidemiological and behavioural surveillance system to inform planning and priority setting, and combat non-communicable diseases and their risk factors, including tobacco use [28]. Similarly, Egypt established an Epidemiology and Disease Surveillance Unit at the Ministry of Public Health in 2000 but data generation and sharing is “*seriously fragmented*” [29].

#### Access to resources

A lack of resources “*is often the excuse for not conducting the required studies and research*” in the EMR according to an article from a tobacco expert at EMRO [30]. Additionally, it was noted that when resources are available the preference is to allocate these for activities which result in immediate outcomes such as media campaigns, rather than data collection [30].

### Development and formulation of policy

#### Evidence base

A recurring barrier to general tobacco control policy development in the EMR is the lack of an appropriate evidence base. It was stated in multiple publications that the lack of evidence generated in Egypt, Iran, and the wider Arab world is hindering the development of effective tobacco control policy [21, 23, 24, 28, 30]. The process of policy development for tobacco control is particularly in need of evidence regarding the evaluation of previous policies and programmes, with tobacco control experts in Iran suggesting the evaluating the efficacy of interventional programs as a priority [21]. However, it was also stated by Koplan that “*assumptions that research findings will lead to policy change, basing policy on evidence, are overly optimistic*” and “*many governments do not instinctively reach for data when designing policy*” [31].

#### Health system structure

Post Islamic revolution, health research in Iran was modified through the restructuring of medical education and its integration into the health care delivery system, an “*innovative action*” which “*might influence the research priority setting and provide better insight into the importance of health system research*” [32]. Egypt, on the contrary, has been privatising its health services over the past decades and decentralising some health related decision making to regional governorates, which has contributed to fragmented data sharing [29]. Government cohesion is necessary for optimal policy development. A report by The Economist stated that ministers in Egypt have clashed regarding policy development, the finance ministers praising economic boosts from the tobacco industry, while health ministers dismissing short term benefits as little compensation for total health burden costs [33].

#### Tobacco industry influence

The tobacco industry was noted as having employed tactics to prevent and disrupt the development of policy in both countries [34, 35]. It is said by the WHO to have “*influential connections with national decision makers”* in Egypt [35]. An example given in the literature was a leading member of the Egyptian parliament assuring the industry that “*no advertising bans would pass parliament*” though this did happen regardless. In response, the industry lobbied “*against the issuance of an executive order banning advertiseme*nt” [34]. Attempts by the tobacco industry to influence policy in Egypt before and after the 25^th^ January Revolution of 2011 have been documented by the WHO, when the industry “*capitalised on the political and economic upheaval facing the country”* [36, 37]. A qualitative study of health experts in Iran identified the determination of tobacco companies and their impact on national politicians as an important barrier to the formation of tobacco control laws, though this was not addressed elsewhere in the literature [21]. This type of evaluation was not found to have been carried out in Egypt.

#### Religious influence

The multiple occurrences of a religious declaration, or Fatwa, on the banning of smoking in Iran has been cited as a factor by national health experts which has potentially influenced the development of tobacco control policy having being ratified by the government in both 1992 and 1994 [21]. A Fatwa was also declared in Egypt in 2000 against tobacco and while it is claimed that there is a need to use religion “*as a pillar of public health interventions*”, this is not a sufficient argument for using religion “*as a basis of public health policy*”. [38].

### Policy implementation

#### Government coordination

Personnel and sustainable funding were identified by the WHO, EMRO and tobacco control experts in Iran as essential for the implementation of tobacco control policy [20, 21, 30, 35, 39]. The workforce in Egypt is insufficient for enforcing policy, and more people must be trained and employed [35]. A lack of subnational infrastructure and a full political commitment to tobacco control in Egypt is preventing the implementation of policy [34, 35].

#### Knowledge and attitudes

A survey of healthcare professionals and hospital administrative employees found that “*the vast majority of participants (>88%) identified no enforcement of smoke-free policies as a barrier to successful implementation of smoke-free hospital policies*”. The factors which were highlighted as the major obstacles to implementing smoke-free hospital policies were the lack of penalties for violations, the absence of cessation programmes for those willing to quit, and the fact that physicians were smokers [40]. It was suggested that research showing overwhelming positive public opinion for smoke free policies is necessary for success in the implementation of smoke-free policy alongside rigorous procedures to ensure compliance [41]. In Iran a cross sectional survey of dental students found the most important barriers to providing cessation services were “*perceived patient resistance and the lack of a supportive organisation*” [42].

#### Access to primary care

Access to primary health care was stated as an important element of implementing cessation services [20]. Results from the GATS between 2008 and 2011 found that in the last 12 months 21.6% of smokers in Egypt had visited a health-care provider in the last 12 months, the lowest score when compared to 16 other LMIC studied. In the same survey Egypt placed third highest for tobacco users who had been screened by a health-care provider in the last 12 months for tobacco use (74.1%) [43]. This survey was said to indicate that “*opportunities exist globally for health-care providers to screen for tobacco use and provide smokers with advice to quit*”. A suggestion for LMIC was “*optimizing population coverage and using health services, promoting community-based interventions, and developing partnerships with health-care systems to support cessation and treatment*” [43].

#### Tobacco use in the media

A lack of leadership and regulation of the film industry has been cited as being responsible for a “*dramatic and disturbing upward trend in depictions of smoking in Iranian movies from 1982–2011*”, undermining the implementation of legislation banning the promotion of smoking in media [44]. A study carried out in Alexandria, Egypt’s second largest city, found that exposure to Western media, which frames smoking in a glamorous manner, has been shown to have a positive association with smoking risk in adolescence; this is despite advertising through international media in Egypt was banned in 2002 [45].

In a speech given by the UNICEF representative in Iran, it was said that “*purposeful engagement between media and health and avoiding the promotion of harmful products or behaviors that can affect people’s health*”, including tobacco [46].

#### Taxation and cigarette smuggling

The complexity of tobacco tax structure can hinder its implementation. In Egypt a complex eight-tiered specific tax structure was changed to more simplified system intended to increase the ease of implementation [47]. Although tobacco taxation in Iran is very low (<10% of retail price) and the system is not complicated, it faces a significant barrier to implementation due to the consumption of smuggled cigarettes. By the 1980s, multinational tobacco companies began smuggling their products into Iran to “*avoid cost additive taxes levied by a Protectionist Iranian Government*” [48].

#### Use of the water pipe

The increasing use of the waterpipe in Egypt and Iran is presenting challenges for the implementation of tobacco control policy. While the cigarette tobacco industry is well defined, and cigarettes are sold in a similar shape and size of packaging globally, this is not true for the waterpipe. In comparison, the waterpipe is “*variable in shape and size, less portable, and is often shared*”, and policy requires to be adapted accordingly. The widespread use of the waterpipe also poses a challenge to the enforcement of smoke-free policy as establishments where these are consumed are persistently challenging indoor smoking bans [34].

## Discussion

This study found that concerns regarding inadequate surveillance systems and their inability to generate evidence in Iran, Egypt, and the EMR in general were recurrent in the literature [20, 21, 23, 24, 28, 30]. While multiple institutions conducting research relating to tobacco were identified, the effect of these was not determined. Internationally standardised surveys were found to be not conducted frequently enough to collect adequate data, and in instances where they are carried out, there may be issues with the internal validity of data [24, 28]. While the lack of an evidence base was given as a reason for causing the delay in policy development in Iran and Egypt, there is an abundance of data collected as part of the Global Tobacco Surveillance System surveys from the WHO and other studies, which could be applied to policy making, as well as multiple institutions researching tobacco related issues. It was also noted that a lack of resources may hinder research studies, while data sharing was said to be fragmented in Egypt due to the privatisation of health services and decentralisation of decision making [29, 30]. This poses the question of whether it is political will rather than lack of evidence which is slowing the development of policy. The example of rapid policy development and implementation in Turkey, following high level decisions to contain the tobacco epidemic, shows the potential of political commitment [49]. Though Turkey is a country where tobacco consumption is a deeply embedded social norm, the passing of numerous tobacco control laws have led to decreased tobacco use and have earned international recognition [50]. From Iran’s efforts to combat tobacco consumption it could be confidently said that their government is committed to the FCTC, it being the only EMR country to achieve the highest rating for 5 of the 6 measures developed by the FCTC to monitor efforts [51]. Due to the disruptive period of political transition from 2011–2014 in Egypt, it is understandable that the government may not be as cohesive as in Iran.

Multiple literature sources have identified the tobacco industry as a major barrier to the development of policy in both Egypt and Iran. It was stated that the tobacco industry has repeatedly tried to restrict tobacco control activities in Egypt as early as 1981 through lobbying and infiltrating parliament [36]. Information regarding the industry’s influence in Iran was scarcer, but featured in a qualitative study of health experts in Iran [21]. Religious influence was cited as a possible influence on tobacco control policy in Iran, but its effect on policy development was not conclusive [21].

Despite the implementation of warning labels for cigarette packages in Iran, it was suggested several times that the widespread trade of smuggled cigarette packages has affected the implementation. However, with the removal of international sanctions in 2015 which were imposed on Iran, the nature of international trade with the country is set to change with possible ramifications for the tobacco trade. A decrease in smuggled cigarette usage could have further benefits on revenue from tobacco taxation. The potential of using cigarette taxes to decrease consumption is shown in Brazil, where almost half of the 46% reduction in smoking prevalence from 1989–2010, was due to the increased cost of tobacco [52]. This is of particular note as taxation is one of WHO’s “best buy” interventions against NCD in LMIC due to its effectiveness and economic viability [11, 53].

While information from the literature suggests that tobacco control implementation is poor in Egypt due to lack resources for enforcement, two surveys carried out by the WHO to quantify the extent of FCTC implementation offer a different view [12, 54]. Out of the 22 EMR countries, Egypt was rated second after Iran in both surveys, with an increase in compliance to smoke-free policy from 2011–2013 [12]. Indoor smoking bans are, however, challenged by establishments where tobacco is consumed using the water-pipe, which is increasing popular among youth and female populations where tobacco use was traditionally not prevalent [34, 55]. The availability of primary care was identified as an important factor for enabling access to services for cessation and controlling the burden resulting from tobacco use in the EMR [43]. However, it must also be considered that it may take the implementation of policies for smoke-free areas, warning labels, and taxes to create demand for cessation services. Healthcare professionals require support in providing cessation services and changing the attitudes of patients and the public, with the depiction of tobacco use in the media in need of addressing, though bans on tobacco use in the media exist in both Egypt and Iran [41, 44].

The stark difference in financial resources and personnel devoted to tobacco control must be considered when comparing the countries, with per capita expenditure at over 53 times higher in Iran, which is crucial to the country’s ability to conduct research and implement policy. The annual budget for tobacco control in Egypt rose from $12,500–30,000 in 2009 and has stayed constant since [1, 51, 56–59]. This figure differs substantially from the $2 million annual budget in Iran which was in place from 2008 till 2011, while the budget dipped to $500,000 per year for the next two years it rose again to $1,500,000 in 2014 [1, 51, 56–58]. The number of full time staff working for the national tobacco control programme in Iran has been 20 since 2008 and 3 since 2008 in Egypt [1, 51, 59]. Egypt ranks below fellow middle income countries Vietnam and India, and even low income countries Bangladesh and Nepal, despite having the largest GDP per capita [60]. It should be noted also that tobacco control expenditure is self-reported, which may increase the risk of bias.

This is the first comparison of its kind between two such countries, both prominent in the fight against tobacco consumption in the EMR. This study builds on previous research which has focused on comparing the status of policy and impact by investigating the influencing factors. The general topic of the tobacco control policy process has been researched in a variety of manners, including policy analysis triangle, qualitative case studies, and literature review. Investigations the factors facilitating and hindering the policy process have been completed in several European countries using a policy triangle for analysis [61, 62]. Many of the factors covered are applicable to other LMIC, both within the EMR and further afield, particularly as the tobacco industry is aggressively expanding their markets in these countries. A literature review of the political economy analysis of implementing tobacco control in LMIC identified five policy areas as targets for further policy economy analysis. These included the use of smuggling, awareness of dangers in using tobacco among citizens, incentive conflicts in government, barriers to raising taxes and establishing smoke-free space, and the roles of tobacco producers and trade disputes in consumption [63].

The scoping nature of the study is intended to identify and synthesise all of the literature published thus far on this multi-faceted area. In particular, this study complements two papers published by Heydari in 2012 and 2014 by using literature sources to investigate the reasons behind the differing levels of implementation and policy development [12, 54]. While there is merit in reviewing the available literature, it is important to conduct qualitative research with stakeholders in the region and similar settings to develop and solidify recommendations and provide an up to date account of the current situation. This is in line with the additional optional step of scoping review methodology: consulting with stakeholders to provide insights on what is beyond what it is possible to extract from the literature.

### Limitations

Due to the nature of scoping review methodology, references are not assessed for quality or risk of bias, as the aim was to assimilate as much literature that had been published on the subject as possible, due to the paucity of this. A wide variety of study designs is both a positive and negative attribute as gives more width than depth to the findings. As this study was limited to health and medical related literature databases, it does not encapsulate the policy process in its entirety, particularly the social and political science aspects of tobacco control policy. Understanding the wider political context surrounding the policy process is key to resolving hindrances, and future research should take into consideration studies which focus on the social and political factors involved in the process of using policy for tobacco control. While issues have emerged in the literature which need to be addressed, more qualitative research requires to be carried out in the likes of Egypt to gain a deeper understanding of current failings in using tobacco control policy. Transferring the FCTC into national policy requires thorough consideration of contextual influences - the process of which has not been explored in this review but is a key element of ensuring policy is appropriate for each country. The inclusion of review and commentary articles as well as primary research was in accordance with the principles of a scoping review which aims to collate all types of literature associated with a research aim, however this potentially lessens the ability to draw concrete, balanced conclusions.

## Conclusions

Iran is seen to be succeeding in using policy to control tobacco consumption to a greater extent than Egypt through the strengthening of tobacco research and surveillance, the development of evidence based policy, and its implementation. This is facilitated by the Iranian government’s greater commitment to tobacco control as demonstrated by the devotion of more resources towards the enforcement of policy and push to deliver cessation treatment through primary care. While Egypt is lacking on these points, it is showing encouraging progress towards using taxation to reduce tobacco consumption, which should be noted by Iran.

Multiple factors were identified as affecting the process of using policy for tobacco control such as the lack of an evidence base for development, poor political commitment to implementation, lack of resources for evidence generation, government cohesiveness, tobacco industry influence, and the smuggling of cigarettes. Several factors arose which have not been investigated in depth for Egypt and Iran, such as the influence of the tobacco industry, and developing policy to address water pipe use.

Egypt should assess the transferability of implementation tactics in Iran and use accordingly. Egypt should also address its very low expenditure on tobacco control by earmarking a higher proportion of tax earned from tobacco products – a straightforward method which could have very positive repercussions for the ability to enforce policies such as smoke-free legislation. Both countries are lacking in international standardised nationally representative data and the repeated implementation of WHO recommended surveys should be prioritised.

## Appendix

**Table 3:** Full search terms with results

Search	Search terms	Embase	MEDLINE	Web of Science	PubMed	Global Health
1	Egypt* and tobacco and (policy or policies)	30	14	41	27	13
2	Iran* and tobacco and (policy or policies)	31	27	61	31	26
3	Egypt* and tobacco and research	36	66	68	99	17
4	Iran* and tobacco and research	70	99	144	308	50
5	Egypt* and tobacco and (policy or policies) and develop*	9	5	20	2	13
6	Iran* and tobacco and (policy or policies) and develop*	9	5	25	4	25
7	Egypt* and tobacco and implement*	20	9	23	2	10
8	Iran* and tobacco and implement*	27	24	53	5	31
9	Egypt* and tobacco and (adherence or compliance)	6	3	5	4	2
10	Iran* and tobacco and (adherence or compliance)	8	4	8	7	3
11	Egypt* and tobacco and regulation	3	1	8	14	1
12	Iran* and tobacco and regulation	9	2	8	17	1
13	Egypt* and tobacco and legislation	5	3	13	12	5
14	Iran* and tobacco and legislation	2	2	11	7	3
15	Egypt* and non-communicable disease and (policy or policies)	5	1	32	8	1
16	Iran* and non-communicable disease and (policy or policies)	14	9	127	60	5
17	Egypt* and (smok* or cigarette* or water?pipe) and (policy or policies)	34	17	44	31	14
18	Iran* and (smok* or cigarette* or water?pipe) and (policy or policies)	70	50	111	63	33

**Table 4:** Records Identified Through Literature Search

Title	Author	Year	Journal	Design	Policy research	Policy development	Policy implementation
Tobacco cessation practices of senior dental students in Iran	Ahmady A., et al.	2011	International Dental Journal	Cross-sectional			X
Sanctions, smuggling, and the cigarette: The granting of Iran office of foreign asset control's licenses to big tobacco	Batmanghelidj E., and Heydari G.	2014	International Journal of Preventive Medicine	Systematic review			X
Health-care provider screening for tobacco smoking and advice to quit - 17 countries, 2008–2011	Caixeta R., et al.	2013	Morbidity and Mortality Weekly Report	Report using GATS data			X
Tomorrow’s regular customers? Stamping out tobacco use in the Middle East and Africa	Economist Intelligence Unit	2009	NA	Report		X	
Tobacco control in the Eastern Mediterranean Region: overview and way forward	El Awa, F.	2008	Eastern Mediterranean Health Journal	Review	X	X	X
Establishment of respiratory disease network in Iran	Ghanei M., et al.	2011	Tanaffos	Editorial	X	X	
Time trend of smoking scenes in Iranian movies during the past three decades (1982–2011): a historical analysis	Heydari G., et al.	2015	Tobacco Control	Historical film analysis			X
Western media's influence on Egyptian adolescents' smoking behaviour: the mediating role of positive beliefs about smoking	Islam S., et al.	2007	Nicotine & Tobacco Research	Cross sectional study			X
Egyptian Smoking Prevention Research Institute (ESPRI).	Israel E., et al.	2003	Journal of the Egyptian Society of Parasitology	Commentary	X		
Religion-based tobacco control interventions: how should WHO proceed?	Jabbour S., and Fouad M.	2004	Bulletin of the World Health Organization	Commentary		X	
Curtailing tobacco use: First we need to know the numbers	Koplan J., and MacKay J.	2012	The Lancet	Commentary		X	
Meeting report on first national seminar of media and health: 7th August 2014, Shiraz, Iran	Lankarani K., et al.	2014	Shiraz E Medical Journal	Meeting report			X
Health status, epidemiological profile and prospects: Eastern Mediterranean Region	Mandil A., et al.	2013	International Journal of Epidemiology	Review	X	X	X
Joining the coalition: Gard, Iran.	Masjedi M.	2008	Tanaffos	Editorial	X		
Tobacco in the Arab world: old and new epidemics amidst policy paralysis	Maziak M., et al.	2013	Health Policy and Planning	Review		X	X
Health research priority setting in Iran: Introduction to a bottom up approach	Owlia et al.	2011	Journal of Research in Medical Sciences	Priority setting		X	
Public opinion on smoke-free policies among Egyptians	Radwan G., et al.	2012	International Journal of Tuberculosis and Lung Disease	Cross sectional study			X
Implementation, barriers and challenges of smoke-free policies in hospitals in Egypt	Radwan G., et al.	2012	BMC Research Notes	Cross sectional study and hospital inspection			X
Non-communicable diseases in the Arab world	Rahim H., et al.	2014	The Lancet	Review	X	X	X
Evidence for Policy in Iran	Sadrizadeh B.	2009	Iranian Journal of Public Health	Commentary		X	
The path towards universal health coverage in the Arab uprising countries Tunisia, Egypt, Libya, and Yemen	Saleh S., et al.	2014	The Lancet	Lancet Series Review		X	
A Comprehensive Model to Evaluate Implementation of the World Health Organization Framework Convention of Tobacco Control	Sarrafzadegan N., et al.	2012	Iranian Journal of Nursing and Midwifery Research	Qualitative study	X		
Indicators developed to evaluate the international framework convention on tobacco control in Iran; a grounded theory study	Sarrafzadegan N., et al.	2014	Iranian Journal of Medical Sciences	Qualitative study	X		
Health experts' opinions about tobacco control activities in Iran: Results from a delphi panel of national experts	Sharifi H., et al.	2012	Tanaffos	Qualitative case study	X	X	
The tobacco industry's tactics and plans to undermine control efforts in Egypt and North Africa	World Health Organisation	2003	WHO	WHO report		X	
Progress in tobacco control in Egypt and Pakistan: activities implemented by WHO under the Bloomberg initiative to reduce tobacco use	World Health Organisation	2010	WHO	WHO report	X	X	
Tobacco industry tactics and plans to undermine tobacco control efforts	World Health Organisation	2012	WHO	WHO report		X	

## Abbreviations

EMR: Eastern Mediterranean Region
EMRO: Eastern Mediterranean Regional Office
ESPRI: Egyptian smoking prevention research institute
FCTC: Framework convention on tobacco control
GATS: Global adult tobacco survey
GDP: gross domestic product
GSPS: Global school personnel survey
GSPSS: Global health professions student survey
GYTS: Global youth tobacco survey
IrEA: Iranian epidemiological association
LMIC: low and middle income countries
NCD: non-communicable disease
STEPS: STEPwise approach to surveillance
WHO: World health organization
